# Genetic and Physiological Factors Affecting Human Milk Production and Composition

**DOI:** 10.3390/nu12051500

**Published:** 2020-05-21

**Authors:** Yarden Golan, Yehuda G. Assaraf

**Affiliations:** The Fred Wyszkowski Cancer Research Laboratory, Department of Biology, Technion-Israel Institute of Technology, Haifa 3200003, Israel; golanyar@campus.technion.ac.il

**Keywords:** human milk, nutrition, nutrigenomics, nutrigenetics

## Abstract

Human milk is considered the optimal nutrition for infants as it provides additional attributes other than nutritional support for the infant and contributes to the mother’s health as well. Although breastfeeding is the most natural modality to feed infants, nowadays, many mothers complain about breastfeeding difficulties. In addition to environmental factors that may influence lactation outcomes including maternal nutrition status, partner’s support, stress, and latching ability of the infant, intrinsic factors such as maternal genetics may also affect the quantitative production and qualitative content of human milk. These genetic factors, which may largely affect the infant’s growth and development, as well as the mother’s breastfeeding experience, are the subject of the present review. We specifically describe genetic variations that were shown to affect quantitative human milk supply and/or its qualitative content. We further discuss possible implications and methods for diagnosis as well as treatment modalities. Although cases of nutrient-deficient human milk are considered rare, in some ethnic groups, genetic variations that affect human milk content are more abundant, and they should receive greater attention for diagnosis and treatment when necessary. From a future perspective, early genetic diagnosis should be directed to target and treat breastfeeding difficulties in real time.

## 1. Introduction

### 1.1. Hormones Inducing and Sustaining Human Milk Production: Prolactin and Oxytocin

The mammary gland is a unique glandular organ that undergoes major morphologic and physiologic changes during the life of the organism. During puberty, hormones including estrogen initiate mammary gland development to support lactation [1,2,3]. Later, during pregnancy, elevated levels of progesterone and prolactin initiate the alveolar development in the mammary gland as well as the proliferation of mammary gland epithelial cells [4]. The lactogenic hormones estrogen, progesterone, and prolactin and metabolic hormones such as insulin are required for the differentiation of mammary epithelial secreting cells, which are capable of synthetizing and secreting specific milk components. This process is termed “secretory differentiation” and it continues during pregnancy [5,6]. After delivery, hormonal changes initiate milk secretion from mammary gland epithelial cells, a stage termed “secretion activation”. In this stage, the levels of progesterone decrease dramatically, whereas prolactin, insulin, and cortisol are essential for regulation of this crucial phase [5,6]. In addition, oxytocin, a nine amino acids neuropeptide hormone that is synthesized in the hypothalamus, is secreted by the posterior pituitary to the circulation as a trigger for infant suckling the mother’s nipple [7,8,9]. Oxytocin is responsible for the induction of contraction of myoepithelial cells of the mammary gland, resulting in milk ejection [7], or the “let-down” reflex that releases the milk from the ducts [8]. In this respect, oxytocin-deficient mice failed to lactate their pups, and this important function of oxytocin could not be compensated by other mechanisms [8]. The complex hormonal regulation of breast milk production and secretion may largely affect the outcome of lactation. However, very little is known about how genetic variations in these key factors for lactation, affect human breastfeeding success.

### 1.2. Physiological Changes in Breast Milk Composition

Breastfeeding has numerous positive impacts on infants beyond the nutritional value of milk. Human milk is the best nutrition for infants during the first six months after birth and it is highly recommended to continue breastfeeding along with supplementary foods up to two years of age [10]. The composition of the transitional and mature human milk differs largely from the colostrum, which is secreted in the first days of lactation and contains many immunologic components [11]. The concentration of some human milk components including vitamins B_6_, B_12_, and vitamin C (ascorbic acid) declines with the duration of breastfeeding, until weaning [12]. Furthermore, calcium, iron, zinc, and copper concentrations gradually decrease throughout lactation [13]. Additionally, the concentration of some nutrients in the milk depends on the mother’s diet [14,15]. In most cases, the transport of nutrients to the milk will continue even upon maternal deficit, and milk prediction may lead to reduction of the mother’s reserves. However, in some cases of inadequacy of maternal diet, the infant may experience inadequate levels of micronutrients, which might be critical for the infant’s health [16]. Vitamin B_12_ levels were shown to be in correlation to the mothers’ diet, with lower concentrations in milk of vegetarian mothers compared to omnivorous mothers [17]. Vitamin K as well as vitamin D concentrations in human milk were found to be non-sufficient, thus they should be added to the infant’s diet right after birth [18,19]. With the current introduction of new analytic techniques for the reliable quantification of nutrient levels in human milk [20], our knowledge about the normal variation and adequate levels of the various components in breast milk will increase, towards the optimization of the nutritional status of the infants. The physiologic decrease in some human milk components is reasonable when taking into consideration that infants are able to consume other food types when reaching the age of ~6 months. Therefore, the mother’s milk is not the only nutritional source for the infant at this age, and the infant is supposed to obtain the deficient nutrients from supplementary foods.

## 2. Materials and Methods

The aim of the present review paper was to summarize the genetic variations found to affect human milk production or content. An Internet search using Google Scholar and PubMed databases was used to identify and download abstracts and papers related to this topic using various key terms, including genes/genetic/variations/mutations/single nucleotide polymorphism (SNP) and human milk/breast milk.

## 3. Genetic Variations that Affect Human Milk Composition

In addition to maternal nutrition and hormonal regulation that may affect both breast milk volume and composition, certain genetic variations were recently reported to affect human milk composition [21,22,23]. Table 1 depicts the summary of variations that were associated with changes in human milk components. These genetic variations are detailed in the following section.

### 3.1. ZnT2 (SLC30A2) Mutations and Transient Neonatal Zinc Deficiency (TNZD)

In 2006, the first mutation in the *SLC30A2/ZnT2* gene was found to be associated with transient neonatal zinc deficiency (TNZD), a disorder that leads to severe zinc deficiency exclusively in breastfed infants [24]. Since this pioneering report, at least 10 distinct mutations in *SLC30A2/ZnT2* were identified in mothers producing zinc-deficient milk, and consequently in the blood of their exclusively breastfed infants, which developed severe zinc deficiency [25,26,27,28,29,30,31,32,33]. This disorder was termed TNZD, as the symptoms associated with zinc deficiency resolved upon zinc supplementation of the infants during the lactation period and these infants were completely cured after weaning. We have recently proposed that a haploinsufficiency state occurs in women harboring heterozygous mutations in SLC30A2/ZnT2, which implies that a single gene copy of the wild type (WT) allele is not sufficient for the secretion of zinc into the milk to meet the infant’s nutritional needs [32]. We have further reported that the frequency of individuals harboring TNZD causing alleles was as high as 1 in 2334 individuals from various ethnic groups [34]. Furthermore, the frequency of the specific founder p.G87R mutation in the Ashkenazi Jewish population was even higher and attained a striking frequency of 1 in 576 individuals (unpublished data); this frequent mutation is known to cause TNZD [29]. In this respect, we previously discovered that the heterozygous G87R mutation inflicts a dominant negative effect on the WT ZnT2 allele via dimerization, hence disrupting its zinc transport activity [29,35].

Additionally, although ZnT2-KO mice display only a modest reduction in the milk zinc content (~30% reduction), presumably due to the compensatory transport function of ZnT4, major deleterious effects were reported on both mammary gland development and function as well as on milk volume and composition including protein, fat, and lactose content during lactation [36]. If these findings also reflect the function of ZnT2 in women’s mammary gland, it may negatively affect not only human milk composition, but also the actual breastfeeding ability. Importantly, these findings might explain the relatively low number of TNZD cases that were reported in the literature in contrast to the high frequency of TNZD causing alleles in the general population [34]. If women harboring loss of function (LoF) mutations in *SLC30A2/ZnT2* also suffered from low milk supply, supplementary foods must be provided to the infant relatively early (i.e., baby formula) which will therefore mask the zinc deficiency in the milk. In addition to LoF mutations, some single nucleotide polymorphisms (SNPs) were identified in *SLC30A2/ZnT2*. In one case, the L23P SNP was associated with low zinc levels in human milk [37]. Owing to the higher frequency of SNPs in some ethnic groups, it is highly important to investigate their effect on breast milk zinc concentration in order to prevent deficiencies in the nursing infants. We recently established a genetic test for the detection of maternal *SLC30A2/ZnT2* mutations, using RNA from cells that are present in the mother’s milk, for early diagnosis and prevention of TNZD [32]. To date, infants are diagnosed only after they show symptoms of severe zinc deficiency, which include severe erythematous scaly rash involving the face, neck, and diaper area. However, asymptomatic cases of moderate zinc deficiency are undiagnosed and untreated [32]. The recommended treatment for TNZD was zinc supplementation (5 mg/day) together with continuing breastfeeding until weaning [32].

### 3.2. ABCG2 (BCRP)—Riboflavin Secretion and Milk Volume

The gene expression of the ATP-driven, multidrug efflux transporter, breast cancer resistance protein (BCRP/ABCG2) was shown to be strongly induced in the mammary gland during pregnancy and lactation in mice, cows, and humans [38]. In mice, knock-out of *BCRP1* lead to a 63-fold reduction in riboflavin (vitamin B_2_) concentration and 3-fold reduction in biotin levels in the milk [38]. Interestingly, heterozygous mice pups (*Bcrp1*^+/−^), which were nursed by *Bcrp1*^−/−^ dams, showed only a modest reduction in their plasma riboflavin concentration 7–12 days after birth, probably owing to conversion of flavin adenine dinucleotide (FAD) (which was found in normal levels in milk of *Bcrp1*^−/−^ dams that were fed with normal diet), the enzyme cofactor of riboflavin [38]. Hence, two distinct transport systems are apparently operating in the lactating breast epithelium, which secrete both riboflavin and its enzyme cofactor FAD. In contrast, pups nursing from WT or *Bcrp1*^−/−^ dams consuming a riboflavin-deficient diet were reported to suffer from riboflavin-deficiency symptoms such as growth retardation, fatty degeneration of liver, and severe anemia [38]. These observations are in accordance with the importance of riboflavin for infant development, which makes it reasonable to preserve two independent pathways for riboflavin transport into the milk. Consistently, in Holstein cattle breed, genetic variations in *ABCG2* were associated with milk yields and fat as well as milk protein percentage [39,40]. In women, the *ABCG2* c.421C > A polymorphism was shown to affect the export of nifedipine (a calcium channel blocker used to treat hypertension during breastfeeding) to the milk [41]. This SNP, which is highly abundant in different ethnic groups, was shown to alter the activity of ABCG2, leading to significant changes in nifedipine pharmacokinetics [41]. It was specifically shown that mothers harboring the heterozygous c.421C > A polymorphism have a significantly higher milk to plasma nifedipine ratio than mothers harboring the c.421CC genotype. Further research is needed to evaluate the effect of this variant and other genetic variations in *ABCG2* on riboflavin concertation in human milk and how a maternal diet may affect the riboflavin concentration in the presence of these SNPs. Additionally, these results show that genetic variations in transporters, which are highly expressed in the mammary gland during lactation, such as ABCG2, affect the pharmacokinetics of drugs to the milk, and emphasize the importance of understanding the effect of these variations not only on the transport of nutrients, but also on drug delivery into the milk.

### 3.3. Genetic Variations in the Sodium Iodide Symporter (NIS/SLC5A5) and Iodine Deficiency in Human Milk

Iodine uptake from human milk is crucial for the production of iodine-containing thyroid hormones and, consequently, normal cognitive and physical development of infants [42]. Specifically, thyroid hormones are required for proper neuronal development [43,44,45]. Iodine concentration in human milk depends on the maternal iodine status, and it was reported to increase with iodine supplementation or consumption of iodized salt [46]. However, iodine concentration is 20–50-fold higher in human milk when compared with its plasma concentration [46]. It is also thought that it might be transported to the milk even under maternal iodine deficiency, owing to depletion of maternal reserves [42]. Iodine is actively transported into the mammary gland epithelial cells, mainly by the sodium iodide symporter (NIS/SLC5A5), the expression of which is upregulated in the mammary gland during lactation [42]. To the best of our knowledge, no iodine exporter was reported to be expressed in the human mammary gland during lactation and the mechanism of iodine transport from the cytoplasm of mammary gland epithelial cells into the milk remains to be established. RT-PCR analyses in the mammary glands of lactating mice showed the expression of multiple Ca^++^-activated Cl^−^ channels (CaCC) including TMEM16A, BEST1, and BEST3 [47]. Remarkably, lactating mouse epithelial cells displayed an anion permeability in the sequence: I^−^ > NO_3_^−^ > Br^−^ > Cl^−^ >> glutamate; thus, such CaCC could readily function as efflux transporters of iodine into the milk. In addition, iodine was shown to exit the apical membrane through Pendrin, Ano1, and CFTR to the milk in mice [48]. Looking at the gene expression database from human milk fat globules [49], the iodine exporters that are expressed in this database during lactation are TMEM16A and at very low levels BEST1, suggesting these genes as candidates for iodine to human milk (see gene expression table in [47]). Notably, NIS is expressed in the mammary gland only late in pregnancy and during lactation [50]. Lactation is critical in mammals because maternal milk is the only source of iodine for the newborn. Importantly, when non-lactating mouse dams were treated with oxytocin, their mammary tissue accumulated significantly more radioiodide because NIS expression was induced. In contrast, ovariectomized mice required oxytocin, estradiol, and prolactin to express NIS in their mammary glands [50].

NIS functions as a sodium-iodide co-transporter, exploiting the large blood sodium gradient to drive a concentrative iodide uptake [51]. Iodide transport defect (ITD), a rare autosomal recessive condition that leads to congenital goiter or hypothyroidism, may be a result of homozygous or compound mutations in the NIS transporter. To date, 14 ITD missense and nonsense mutations have been reported that affect the NIS coding sequence (Table 1) [51]. In addition, one mutation in the 5′ untranslated region (a C→T transition at position-54) has also been reported [52]. Recently, a woman carrying the abovementioned homozygous T354P mutation in the NIS transporter was reported to produce iodine-deficient human milk [53]. In this case, the mother was treated with a daily dose of 50 mg potassium iodide starting on the fifth day postpartum until six weeks postpartum, when breastfeeding was ceased [53]. Further research is needed to assess the impact of heterozygous mutations or other genetic alterations in the NIS transporter, which do not result in ITD, and thus are not diagnosed in most cases, based on human milk iodine concentration.

### 3.4. Human Milk Choline Concentrations

Although not a vitamin, choline is an essential nutrient for infants as it is required for their rapid growth and development [74,75]. Choline is essential for many physiological functions. For example, choline is part of the neurotransmitter acetylcholine, as well as the phospholipid phosphatidylcholine, which is a major lipid component in membranes [76]. *MTHFD1* encodes for a protein that has three distinct enzymatic reactions in the interconversion of one-carbon derivatives of tetrahydrofolate, which are substrates for methionine, thymidylate, and *de novo* purine syntheses [77]. SNPs in *MTHFD1* were shown to increase choline concentration in human milk [22]. Using cultured mammary tissues derived from 12–14 day pregnant mice, the impact of the three primary lactogenic hormones prolactin, insulin, and cortisol on choline uptake and incorporation into phospholipids was determined [78]. Insulin alone or in the presence of cortisol and/or prolactin was the only hormone that enhanced choline accumulation in aqueous tissue fractions. In contrast, when present with insulin and cortisol, prolactin was the only lactogenic hormone that stimulated the incorporation of choline into the lipid fraction of mammary tissues. Furthermore, choline uptake was found to be sodium- and time-dependent. Collectively, these findings suggest the existence of a sodium-dependent active transporter for choline in the mouse mammary gland that is specifically stimulated by cortisol, whereas prolactin appears to only stimulate the incorporation of choline into phospholipids [78]. Further studies are required to better characterize the transport mechanism of choline into human milk.

### 3.5. Folate Content in Human Milk

Reduced folates are essential as one-carbon donors for DNA, RNA, and protein synthesis. Folate receptor 1 (*FOLR1*) was found to be one of the highly expressed genes during lactation in human milk cells [79], and was suggested to be a regulator of milk protein synthesis. In goats, *FOLR1* polymorphism was associated with milk production traits [80], suggesting a possible role for this gene in milk production. Although homozygous mutations and SNPs in *FOLR1* were reported in the literature to cause progressive ataxia and myoclonic epilepsy and elevated levels of homocysteine, respectively, the role of heterozygous mutations or SNPs in *FOLR1* on human milk production and content remains to be established. However, studies have established a linear relationship between milk folate concentration and soluble folate binding protein (FBP), currently known as soluble folate receptor (FR) [81]. It should be noted that the transport of folate from the plasma to the milk occurs against a steep concentration gradient (up to 14-fold); therefore, it has been originally proposed that this FBP, which is abundant in human milk, plays a role in folate trapping for secretion into the milk [81]. Although the significance of FBP in milk is not fully revealed, cumulative evidence supports the notion that it is essential for folate secretion, possibly via exocytosis, as occurring with various milk proteins [82]. The presence of folate as a high affinity (Kd = 10^−9^–10^−10^ M) bound species to FBP certainly stabilizes folate, prevents its accessibility to metabolic enzymes, and may enhance its bioavailability to infants [82]. Indeed, original *in vivo* studies have shown that, in contrast to free folic acid, the complex folic acid–FBP was avidly absorbed in the ileum, suggesting a mechanism of intestinal absorption that is distinct from the canonical intestinal folate absorption mediated by the proton-coupled folate transporter (PCFT/SLC46A1) [83,84].

Regarding folate concentration in human milk, in a Canadian cohort study, five folate-related SNPs—*MTHFR* 677C > T (rs1801133), *MTHR* 1298A > C (rs1801131), *MTHFR* 1793G > A (rs2274976), *MTR* 2756A > G (rs1805087), and *MTRR* 66A > G (rs1801394)—were examined for their effect on folate concentration in mother’s milk [85]. None of these SNPs were associated with total folate concentration, however, the *MTHFR* 677C > T SNP was associated with higher levels of unmetabolized folic acid in human milk [85].

### 3.6. Fat Percentage in Human Milk

Milk fat is the major energy source in the infant’s diet [86,87]. Human milk fat composition displays large inter- and intra-individual variation among lactating mothers depending on the duration of lactation, the time during the day, as well as each breastfeeding [88]. However, to the best of our knowledge, genetic variations that affect human milk fat content were not reported to date. In contrast, owing to the high economic importance of fat contents in dairy products, this topic was characterized in depth in dairy animals [12,89,90,91,92,93,94,95]. The diacylglycerol o-acyltransferase 1 (DGAT1) gene encodes for one of the enzymes that covalently attaches diacylglycerol to long-chain fatty acyl-CoAs to form triglycerides [96], which are the major fat type present in milk. Genetic variations in *DGAT1* were highly associated with fat content in dairy animals [89,96,97,98,99,100,101,102,103]. Additionally, these alterations were also associated with milk protein content and milk volume in some species [98,99]. In humans, homozygous recessive mutations in *DGAT1* were found to cause severe congenital diarrhea and protein-losing enteropathy in infants shortly after birth [104,105]. However, the effect of heterozygous mutations on human health and more specifically on human milk production was not yet characterized.

### 3.7. Fatty Acid Desaturases (FADS) and Fatty Acid Composition in Human Milk

*FADS1* and *FADS2* encode for fatty acid desaturases (FADS), which are involved in the biosynthesis of docosahexanoic acid (DHA) from *α*-linolenic acid. Fish and fish oil in the diet are good sources of DHA [23]. Human milk fatty acids were shown to be influenced by the genotype of the FADS1 and FADS2 gene cluster, with significantly lower 14:0, arachidonic acid (ARA), and eicosapentanoic acid (EPA), but higher 20:2 in the minor allele homozygotes of rs174553 (GG), rs99780 (TT), and rs174583 (TT). In contrast, lower ARA, EPA, 22:5, and DHA were found in women carrying the minor homozygous allele G/G of rs174575 [66]. Furthermore, a cohort study (*n* = 309) that examined the effect of three different SNPs in *FADS1* and *FADS2* (*FADS1* rs174561, *FADS2* rs174575, and intergenic rs3834458) on DHA levels in plasma and breast milk l (one month after birth) found lower proportions of DHA in milk from women who were homozygous for the minor allele. This lower proportion could not be compensated for by dietary fish consumption [23]. These findings were later supported by other studies that found that SNPs in the FADS gene cluster influence polyunsaturated fatty acid (PUFA) concentrations in human milk [106,107,108]. Together with a recently published study that found that mothers carrying the minor allele of *FADS1* rs174556 had lower proportions of ARA [108], this finding strongly indicated that genetic variations affect human milk composition. Additionally, genetic variations in FADS and elongase (*ELOVL*) enzymes were shown to affect long chain PUFA (LC-PUFA) levels in human colostrum. Importantly, these variations and human milk LC-PUFA concentration were associated with an advantage in the child’s cognitive ability at the age of 14 months [109]. These findings were later supported by a study on Chinese women that showed correlation between different SNPs in *ELOVL2* and *ELOVL5* and LC-PUFA concentrations [110]. Interestingly, this study found that certain SNP haplotypes together with high dietary intake of DHA increased PUFA levels in human milk when compared with other haplotypes [110]. Higher concentrations of n − 3 long-chain polyunsaturated fatty acids (LCPs), as well as ruminant fatty acids in human milk, showed a protective role in the development of atopic dermatitis (eczema) [111]. These findings highlight the importance of further characterization of genetic variations that affect human milk fatty acid composition that may affect the infant’s nutritional status. These variations may have a long-term effect on the cognition and development of these infants. In these cases, nutrient supplementation of the infant diet may be a possible treatment to enhance infant’s health and cognition.

### 3.8. Human Milk Protein Content

SNPs were suggested to contribute to the normal variations in human milk protein composition [112]. This phenomenon was better characterized in dairy animals, where SNPs in the bovine casein locus were found to be associated with milk yield and milk protein concentration [113,114]; however, very little is known about nucleotide variation and protein content in humans. SNPs in α-lactalbumin were found and characterized in a population containing different ethnic groups, in which no effect was observed on milk *α*-lactalbumin or lactose concentrations [112]. Further research is needed in order to determine if maternal genetic variations in genes such as casein have an influence on human milk protein content.

### 3.9. Human Milk Oligosaccharides (HMOs)

HMOs, a structurally complex and diverse group of glycans, represent the third most abundant component in human milk right after lactose and lipids (excluding water) [115]. HMOs are thought to participate in numerous functions including metabolism, probiotic and prebiotic, antimicrobial, modulation of infant intestinal epithelial cell responses, immune response, nutritional effects, as well as brain development [70,115,116,117,118,119]. Like blood groups, the composition of HMOs depends on the expression of certain glycosyltransferases. On the basis of the secretor (Se) and Le blood group system, which is determined by the activity of two gene loci encoding for the *α*1-2-fucoslyltransferase FUT2 (encoded by the Se gene) and the *α*1-3/4-fucosyltransferase FUT3 (encoded by the Le gene), four milk groups were assigned: Le-positive secretors (*Se^+^Le*^+^), Le-negative secretors (*Se*^+^*Le*^−^), Le-positive non-secretors (*Se*^−^*Le^+^*), and Le-negative non-secretors (*Se*^−^*Le*^−^) [116]. Significant differences in the abundance of total as well as fucosylated HMOs were found in a study that compared the content of HMOs of secretor and non-secretor Chinese mothers [119]. Additionally, the nonsense mutation W143X that introduces a premature stop codon in the fucosyltransferase-2 (FUT2) gene (rs601338), abolished its ability to synthesize *α*(1-2)-fucosylated HMOs and affected the composition of the bifidobacterial population in the infant’s gut [115,120]. In a cohort study that examined HMO levels in Malawian mothers, the total and fucosylated HMO content of milk samples collected from secretor mothers was significantly higher than that of non-secretors [70]. Additionally, two cohorts on Malawian mothers found higher levels of HMOs in the milk of mothers of healthy infants, compared with stunted infants, and these differences were even greater in the group of non-secretor mothers [70]. These findings suggest that HMOs play an important role in infant growth, specifically in undernourished populations. This study highlights the importance of considering the mother’s genetic background in evaluating milk HMOs’ composition. In addition, fucosylated oligosaccharides in the mother’s milk, which depend on the mother’s secretor status, were shown to be highly correlated with the infant gut microbiota diversity and function [71,121,122,123]. The percentage of secretor and non-secretor status in the population may vary widely in different ethnic groups. For example, European and American populations have a non-secretor frequency of approximately 20%, whereas higher frequencies of 32%–38% were found in West African populations [124]. The Canadian Healthy Infant Longitudinal Development (CHILD) cohort study, which enrolled 3624 pregnant mothers from the general population in four major cities across Canada, found that 40% of the Asian population, and 26% of the Caucasian population were non-secretor mothers [125]. Taking into consideration the large number of papers showing a beneficial effect of HMOs on infant growth and development, the genetic variations in different ethnic groups may have a great impact on infant growth, through differential HMOs’ abundance, and further studies in this important domain are warranted.

### 3.10. Other Genes

Cui et al., performed RNA-seq analysis on mammary gland samples from Holstein cows with extremely high and low protein and fat percentage. Using this high throughput analysis, they identified 31 deferentially expressed genes, the expression levels of which were associated with the milk content in these cows [126]. After comparing their data with the genome-wide association study (GWAS) results, they were able to narrow the list of genes to seven candidates that mostly affected milk fat and protein content (*TRIB3, SAA1, SAA3, M-SAA3.2, VEGFA, PTHLH*, and *RPL23A*). These genes were shown to play a role in lipid metabolism (*TRIB3* and *SAA*), secretory functions in the mammary gland (*VEGFA*), as well as mammary gland development (*PTHLH*) [126]. Thus, it will be highly important to test the effect of genetic variations in these genes on human milk composition.

## 4. Genetic Variations that Affect Human Milk Supply

### 4.1. Prolactin

A few cases of familial alactogenesis were reported in the literature [127,128,129,130] as a result of various etiologies. One case with a genetic etiology was identified in a woman carrying a single point mutation in the prolactin gene (*PRL*). This mutation (c.658C > T) was reported to result a premature translation termination, which leads to a marked decrease in the mRNA of this gene via nonsense-mediated RNA decay, hence resulting in familial alactogenesis [131]. The identified LoF mutation in *PRL* was also associated with infertility in this family [131], indicating that women suffering from infertility are more prone to suffer from breastfeeding difficulties and should receive more attention and support in order to succeed in breastfeeding. However, in another case of familial alactogenesis, no mutation in *PRL* was found, indicating that variations in other genes may be associated with alactogenesis. In yet another case, an autoimmune etiology was suggested to cause an isolated PRL deficiency and puerperal alactogenesis in women [129]. These cases emphasize the importance of identifying genetic variations that affect human milk production in order to improve physician diagnosis and reduce women’s stress from breastfeeding failure.

### 4.2. Prolactin Receptor (PRLR)

The prolactin receptor (PRLR) was found to be widely associated with milk yield in dairy animals [132,133,134]. Additionally, *prlr^+/−^* mouse dams showed lactation problems and *prlr* knockout mouse dams failed to display lobuloalveolar development [135]. In humans, a heterozygous LoF mutation (p.His188Arg) in *PRLR* was reported in three sisters with familial hyperprolactinemia [136]. Hyperprolactinemia is determined by higher than normal range levels of prolactin, in which 50% of the cases are caused by tumors [137]. In this case, two sisters suffered from oligomenorrhea and infertility and one sister, between 18 and 31 years of age, had four successful pregnancies with lactation and was treated with dopamine agonist therapy to terminate persistent galactorrhea [136]. The late onset of post-pubertal hyperprolactinemia may explain the differences in the phenotypes between these sisters [136]. However, the fact that one of these sisters, who was successful in lactating her infants, was heterozygous for an LoF mutation in *PRLR* reveals that the latter mutation did not necessarily inhibit or abrogate lactation outcomes. The effect of LoF mutations in *PRLR* on galactorrhea after lactation should be further investigated.

Another case of compound LoF mutations (nonsense R171Ter) and missense (P269L) mutations in the human *RPLR* was recently reported [138]. In this case, the proband’s mother and father were heterozygous for the R171Ter and P269L mutations. The proband suffered from hyperprolactinemia associated with postpartum agalactia, and the mother who carried the heterozygous R171Ter mutation reported insufficient human milk production after her pregnancies, but was successful in breastfeeding when combined with the addition of baby formula until three months post-partum [138]. These findings strongly suggest that homozygous LoF mutations or heterozygous mutations in *RPLR* may have a prominent influence on lactation outcomes and that the prevalence of such mutations in the population should be examined for early diagnosis and support of women that carry these mutations during breastfeeding.

## 5. Discussion

Evolutionary Explanation for Genetic Variation that Affects Human Milk Composition

The ectodysplasin A receptor (EDAR) is responsible for mammary gland ductal branching as well as inducing sweat gland density and incisor shoveling [139]. The EDAR V370A isoform is highly elevated in the North and East Asian populations. It was recently suggested to be a result of positive selection of this allele to increase ductal branching in the mammary gland, thereby enhancing the transfer of critical nutrients to infants through the breast milk during the Last Glacial Maximum [139]. The author suggested that during the Last Glacial Maximum (LGM; 28,000–18,000 years ago), people were living in Arctic Beringia and were exposed to very low levels of UV radiation, which can lead to dangerously low levels of biosynthesized vitamin D [139]. These people experienced selection for polymorphisms in the FADS gene cluster (the encoded enzymes of which are responsible for long fatty acids composition in breast milk) and for *EDAR V370A* because of the advantage of these genetic variants in conferring a better transmission of nutrients from mother to infant through breast milk under conditions of extremely low UV [139].

Another clue for genetic influence on human milk stems from a study that compared the onset of lactogenesis and volume of milk output until the fifth post-delivery day in four ethnic groups: Arabs, Africans, Eastern Europeans, and Italians [140]. The earliest onset of lactation and the highest milk output was registered among Arab and Eastern European women, and they concluded that ethnicity is associated with breastfeeding characterization [140]. These differences between the different ethnic groups may also be related to genetic variations in these populations that evolved under certain environmental conditions in history. One example for such unique ethnic-related SNP is rs35235055 (c.68T > C causing L23P) SNP in *SLC30A2*/*ZnT2*, which is highly frequent in Africans. As mentioned above, this SNP was associated with low levels of zinc in human milk [37,141], and it will be interesting to explore if this SNP had some evolutionary advantage in this specific ethnic group.

In addition to variations in *FUT2* and *FUT3* that were detected at different frequencies in different ethnic groups, as mentioned in Section 3.9 above, other nutrient-related genes were found to be more abundant in some ethnic groups. One example concerns variations in FADS genes, in which approximately 80% of African Americans carry two copies of the G allele at rs174537, which is associated with increased levels of ARA, compared with only 45% of European Americans [142]. These variations may affect individuals’ risk for inflammatory diseases as well as alter their response to dietary interventions [142]. These variations should be taken into consideration upon dietary recommendations setting as well as in breastfeeding and human milk studies. These findings highlight the importance of evaluating genetic variations that are more frequent in specific ethnic groups and offer specific nutritional recommendations based on the identification of genetic variations.

## 6. Conclusions

**Figure 1:**
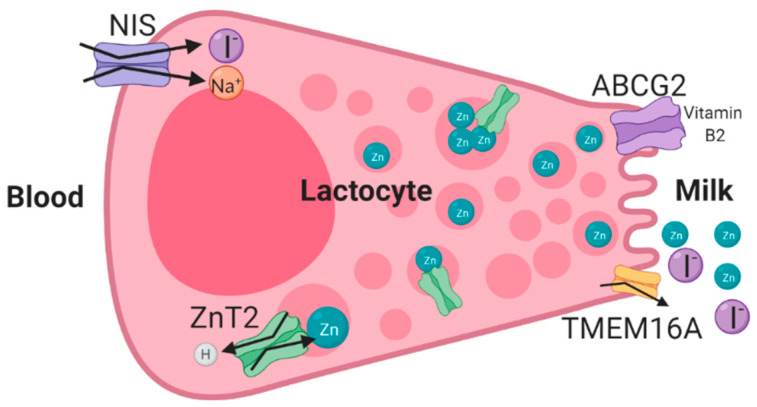
Schematic illustration of the mechanism of transport of micronutrients in human lactocytes during lactation. ATP-driven, multidrug efflux transporter (ABCG2) [38,154], NIS [51,61], and ZnT2 transporter [21,155] were shown to transport riboflavin (vitamin B2), iodine, and zinc, respectively, in human mammary gland epithelial cells during lactation. The anion transporter TMEM16A is predicted to transport iodine into human milk [47,48].

Mammalian breast milk has evolved over millions of years to optimally supply all the necessary nutrients that infants need in order to grow in their first months of life (Figure 1). Production of nutrient-deficient breast milk is against the evolutionary selection, thus genetic variations that diminish breast milk quantity (i.e., volume) and quality (i.e., composition) are relatively rare. However, owing to the use of wet nursing and baby formula, the cases of women who produce nutrient-deficient human milk or who struggle with breastfeeding because of low milk supply, could have been masked. Therefore, genetic variations that affect human milk production, without affecting other physiological functions, may emerge in the population. Furthermore, some of the genetic variations that were discussed above are also associated with infertility. Nowadays, when medical interventions like artificial insemination and *in vitro* fertilization are routinely available, increased attention should be given to the effect of infertility on breastfeeding, and to support these mothers during breastfeeding if needed.

Nutrigenetics and nutrigenomics have been receiving increasing attention over the years, and genetics-based diets are already used [143,144]. Additionally, genetic testing is widely applied for identification of heritable diseases before and during pregnancy [10]. Breast milk is the optimal nutrition for infants in the vast majority of cases; however, in some cases as we described in this article, genetic variations may lead to deleterious changes in milk composition or volume. Maternal genetic tests for candidate genes, some of which were discussed above, and additional genes that will be identified in the future, which may regulate human milk production and composition, may improve maternal and infant health. In cases of nutrient deficiencies like in the case of LoF mutations in *SLC30A2/ZnT2*, which lead to production of zinc-deficient human milk, supplementation of nutrients directly to the infant together with continuing breastfeeding can prevent the disease and improve infant health (Figure 1). In cases of low human milk production, mothers may completely give up on breastfeeding owing to low supply and relatively slow weight gain of the infant. In these cases, mothers that tend to breastfeed and did not reach their own goals may experience failure and depression feelings [10]. However, if the underlying genetic basis could be identified for low milk supply, these women could obtain proper medical attention and support for breastfeeding, and hence continue breastfeeding as long as possible, with less guilt feelings and less stress owing to low milk production. In the future, gene therapy to support breastfeeding may be another option to overcome breastfeeding difficulties.

Personalized medicine implies that the treatment or the drug that is administered to an individual patient should be prescribed based on specific tests or molecular analysis; therefore, the drug should better fit the patient’s disease and lead to improved treatment [145]. One example is drugs that are prescribed based on the patient’s DNA sequence. In recent years, expression of genes such as estrogen receptor (ER) and HER2 has frequently been tested in breast cancer in order to achieve targeted precision medicine [146]. Nowadays, high-throughput technologies such as next generation sequencing (NGS) are used in research and lead to the finding of a high number of heterogeneous gain or loss of function mutations in cancer specimens [146]. However, to translate these research findings into the clinical arena might be challenging and also very expensive, and thus may take a long time to implement [145].

Personalized nutrition (or precision nutrition, nutrigenomics, nutrigenetics, and nutritional genomics) is based on the same principles of personalized medicine, but the treatment is based on the human nutrition instead of drugs or medical treatments [147,148]. Personalized nutrition was recently shown to be very effective in controlling postprandial blood glucose levels [149]. In this study, the researchers developed an algorithm that can predict the postprandial blood glucose levels based on the patient’s microbiome, blood parameters, dietary habits, anthropometrics, and physical activity [149]. In addition, using this algorithm, they were able to successfully generate personalized nutritional recommendation that could lower the postprandial blood glucose levels of the subjects [149]. Other studies used genetics-based analysis, for example, the apoE [150] or amylase [151] genes, to obtain better personalized nutritional recommendation.

Breast milk is the natural version of personalized nutrition. Mothers producing the best food for their infants and milk composition may vary depending on the infants age [19], gender [152], health status [153], and more. In the future, when we will have better insight into the genes that regulate human milk content, we will be able to predict which specific component might be deficient in a certain lactating mother or specific ethnic group, owing to genetic variation/alterations, as we mentioned in this review, and to supplement the infants or the mother diet if needed. These genetic tests will support infant health, growth, and development. Additionally, genetic testing that could determine if the mother is carrying a variation in a gene that may lead to low milk supply could give the medical team an indication that the mother needs more support in order to succeed in achieving her goals of breastfeeding. Furthermore, these tests could reduce mothers’ stress and failure feelings when facing breastfeeding difficulties owing to genetic alterations.

In summary, we herein discussed several deleterious genetic alterations that may lead to significant effects on human milk composition or human milk supply. Further research is warranted in order to identify other genes that regulate human milk production and composition, as well as cellular pathways that are responsible for nutrient transport into human milk. In addition, genetic variations in these genes that affect human milk composition and supply should be further characterized towards the improvement of the health of both the infant and mother.

## Figures and Tables

**Table 1:** Genetic variations that were associated with changes in human milk components, as well as the effect on the infant and suggested treatment. LoF, loss of function; BCRP, breast cancer resistance protein; ABCG2, ATP-driven, multidrug efflux transporter; ITD, iodide transport defect; SNP, single nucleotide polymorphism; HMO, human milk oligosaccharide.

Gene Name	Protein Name	Effect of Mutations on Human Milk	The Effect on the Infant or Related Disease	Treatment
*SLC30A2*	ZnT2	LoF homozygous mutation in ZnT2 were found in mothers producing zinc-deficient human milk [25,26,27,28,29,30,31,32,33].	Transient neonatal zinc deficiency (TNZD), a disorder that leads to severe zinc deficiency in exclusively breastfed infants	Zinc supplementation (5 mg/day) and continuing breastfeeding [32]. No supplementation is needed after weaning.
*ABCG2*	BCRP	Mothers harboring the c.421C > A polymorphism in ABCG2, secreted threefold more nifedipine to human milk [41].	Unknown	
*SLC5A5*	Sodium iodide symporter (NIS)	Mother carrying homozygous T354P mutation in the NIS transporter was reported to produce iodine-deficient milk. Other known LoF mutations in *SLC5A5*: V59E [54], G93R [51], R124H [55], Δ143-323 [56], Q267E [57], V270E [58], C272X [59], Δ287-288 [60], T354P [61], G395R [62], Δ439-443 [63], G543E [64], fs515X [65], and Y531X [65].	The mother was diagnosed with ITD, treated with levothyroxine from the age of five years old, therefore, iodine supplementation was given after birth to prevent deficiencies in the infant.	Mother supplementation with 50 mg potassium iodide tablet daily starting on the fifth day postpartum to increase iodine concentration in human milk.
*MTHFR*	Methylene tetrahydrof-olate reductase	The MTHFR 677C > T SNP was associated with higher levels of human milk unmetabolized folic acid (UMFA) [41].	Unknown	
*MTHFD1*	Methylene tetrahydrof-olate dehydroge-nase 1	rs1076991, rs2983733, rs2987981, rs8003379, and rs17824591 SNPs in the methylene tetrahydrofolate dehydrogenase 1 (*MTHFD1*) gene were found to be associated with very high human milk choline concentrations in three subjects [22].	Unknown	
*FADS1* and *FADS2*	Fatty acid desaturase 1/2	The minor allele homozygotes of rs174553 (GG), rs99780 (TT), and rs174583 (TT) were associated with significantly lower 14:0, arachidonic (ARA, 20:4 ) and eicosapentanoic acid (EPA, 20:5), but higher 20:2 (n − 6) fatty acid in human milk [66]. Mothers carrying the minor homozygous allele G/G of rs174575, showed lower ARA, EPA, and docosahexanoic acids (DHA, 22:6 (n − 3)) and 22:5 (n − 3) levels in human milk [66]. Mothers carrying FADS1 rs174561, FADS2 rs174575, and intergenic rs3834458 minor alleles were found to have lower proportions of DHA in human milk [23].	Mothers carrying genetic variants associated with lower FADS1 activity (regulating AA and EPA synthesis), higher FADS2 activity (regulating DHA synthesis), and with higher EPA/AA and DHA/AA ratios in colostrum showed a significant advantage in cognition at 14 months.	
*FUT2*	Fucosyltra-nsferase 2	Nonsense mutation W143X that introduces a premature stop codon in the FUT2 gene (rs601338) abolished the ability to synthesize *α* (1-2)-fucosylated HMOs (non-secretor status). Non-secretors where found to express less HMOs compared to mothers with secretor status (active FUT2) [67,68,69,70]. In addition, maternal secretor status was shown to be associated with the human milk microbiota composition [71].	Infants fed by non-secretor mothers, were delayed in the establishment of their gut microbiota, specifically *bifidobacterial-laden* [72,73].	
